# Hypertensive patients' perceptions of their physicians' knowledge about them: a cross-sectional study in Japan

**DOI:** 10.1186/1471-2296-11-56

**Published:** 2010-08-02

**Authors:** Machiko Inoue, Kazuo Inoue, Shinji Matsumura

**Affiliations:** 1 Kita-adachi Seikyo Clinic, Tokyo Hokuto Health Cooperative, 3-1-5, Iriya, Adachi, Tokyo, 121-0836 Japan; 2 Department of Community Medicine, Chiba Medical Center, Teikyo University School of Medicine, 3426-3, Anesaki, Ichihara, Chiba, 299-0111 Japan; 3 Matsumura Clinic, 3-4-16, Kaminoge, Setagaya, Tokyo, 158-0093 Japan

## Abstract

**Background:**

In order to evaluate the difference in quality of primary care provided by physicians between the types of medical institutions in Japan, we examined whether the physicians' comprehensive knowledge of their patients is perceived differently by the patients seen at clinics and hospitals.

**Methods:**

Patients with prescriptions for hypertensive drugs were approached sequentially at 13 pharmacies, and were administered a questionnaire on their perception of their physician's knowledge about them. Data were obtained for 687 patients (362 from clinics and 325 from hospitals). A physician's knowledge of his or her patients was assessed according to six aspects: their medical history, their current medications, history of allergy, what worries patients most about their health, patients' values and beliefs on their health, and patients' roles and responsibilities at work, home, or school. Responses were scored from 1 through 6 (1: knows very well; 6: doesn't know at all).

**Results:**

Patients treated in clinics were seen more frequently, for a longer period, and had fewer complications than the patients who were treated in hospitals. Among the six aspects of physicians' knowledge assessed, 79.3% of the patients reported that their physicians knew their complete list of medications "very well or well," while 28.3% reported the same about their roles and responsibilities at work, home, or school. Physicians in clinics were considered to know their patients' worries about their health (p = 0.004) and the roles and responsibilities of the patients at work, home, or school (p = 0.028) well. Multiple regression analysis showed that the type of medical institutions remained as a significant variable only for the aspect of patients' worries about their health. The factor that consistently affected the patients' perception of physicians' knowledge about them was the patients' age.

**Conclusions:**

Hypertensive patients' perceptions of their physicians' knowledge about them did not differ significantly between clinics and hospitals in Japan for most of the aspects. In order to differentiate the roles of physicians in hospitals and clinics better and ensure the quality of primary care, the establishment of a standardized educational system to train primary care physicians better is recommended.

## Background

The provision of primary care and the training of primary care providers in Japan have some unique characteristics [1,2]. First, Japan's universal health insurance system gives a person virtually free access to doctors in any type of medical institution [2,3]. This system allows a patient to choose a specialist in a tertiary care center if he/she wishes for management of symptoms and chronic illnesses that could be managed just as well in primary care centers. As a result, physicians working in hospitals have to see patients presenting with a variety of conditions, and thus carry an excessive patient load [4].

Second, medical education in Japan had traditionally placed little emphasis on primary care training; the reform of postgraduate medical education in 2004 has introduced a two-year compulsory internship [5,6]. Before the reform, most graduates of medical schools trained as subspecialists and worked in university-affiliated hospitals for 5-10 years where they conducted some basic research [7]. Later in their careers, some of them opened their own private clinics [2,3,7-9]. Saigal and colleagues have called this a "Two Career" model of specialization [9], and the inadequacy of primary care training for Japanese physicians has been raised as a critical issue [10-12].

Since the Japanese government launched the health care reform in 2004 to reduce medical costs by differentiating the functions of medical institutions [13], an investigation of the quality of primary care, especially in terms of patient-physician relationship, is merited. With comprehensiveness of care being an important value in primary care defined by the Institute of Medicine [14], the patient-physician relationship in primary care is well characterized in physicians' caring for the patient as a whole person, not merely treating the patient's problems and diseases [15]. We therefore examined whether physicians' whole-person knowledge of the patients with hypertension, which is the most common chronic condition among the Japanese, is perceived differently by patients seen at clinics and hospitals.

## Methods

The study used a cross-sectional observational design. Data were collected as a part of a main study that investigated the difference in patients' knowledge of hypertensive drugs according to the types of medical institutions in which they were prescribed [16]. First, we recruited pharmacies that fill prescriptions from both clinics and hospitals to participate in this study. Thirteen pharmacies in seven cities--Tokyo, Chiba, Saitama, Kanagawa, Nagoya, Osaka and Shiga--agreed to participate. From October to November 2006, patients over 16 years old with prescriptions for hypertensive drugs were approached sequentially at those pharmacies. Those with physical or cognitive disabilities or other conditions who were unable to read and answer the questionnaire in Japanese were excluded. The recruitment of study participants was continued until the number of patients from clinics and hospitals both reached about 400, based on the sample size calculation of the main study, which was intended to investigate the difference in patients' knowledge of the side effects of prescribed drugs in clinics and hospitals (expected difference = 10% (30% vs. 20%), power = 0.90, α = 0.05, a two-tailed test) [16].

The study group developed a questionnaire to assess the patients' perception of their physician's comprehensive knowledge of them according to six aspects (Additional file 1). Four of these aspects were from one of 11 scales of Primary Care Assessment Survey (PCAS) [17]. The scale measures physicians' "whole-person" knowledge of patients; patients' entire past medical history; patients' roles and responsibilities at work, home, or school, what worries patients most about their health; and patients' values and beliefs on health. We added the following two items in order to investigate their drug adherence: the patients' current medications and history of drug and food allergies. Each item was scored on a 6-point Likert scale: 1 = knows very well; 2 = knows well; 3 = knows to some extent; 4 = doesn't know well; 5 = knows little; and 6 = doesn't know at all.

Characteristics of the patient-physician relationship were assessed in two aspects: the frequency of medical visits and the length of the relationship with the physician. The frequency of medical visits had four categories: more than once a month, once a month, once in two months, and once in three months or more. The length of the relationship with the physician also had four categories: less than a year, 1-2 years, 3-5 years, and more than six years. Patients' characteristics were assessed in terms of age, gender, and educational attainment. The status of having or not having each of the following hypertensive complications--retinal hemorrhage/detachments, ischemic heart disease, stroke, and impaired renal function--also was obtained.

All statistical analyses were performed with SPSS version 15.0 for Windows. For baseline data, demographic differences between patients of clinics and hospitals were analyzed by a chi-square test. For each item of characteristics of patient-physician relationships, the difference between clinics and hospitals was assessed, also by using a chi-square test.

Scores for items of the physician's knowledge of patients were dichotomized at the time of analyses to 0 = "doesn't know at all," "knows little," "doesn't know well," and "knows to some extent," and 1 = "knows well" and "knows very well", according to the clinical relevance. A chi-square test was used for each item to evaluate the difference. Statistical significance was determined at p < 0.05.

We conducted a logistic regression analysis to determine the influence of various factors on each item of physicians' knowledge of patients. In addition to the type of medical institutions (clinic/hospital), frequency of visits (<once/≥once in a month), length of the relationship (≥3years/<3years), status of complications (one or more/none) and patient's age (by 5 years) were included as variables because they significantly affected univariate analyses in several items.

This study protocol was approved by the Ethical Committee of the Institute for Health Outcomes and Process Evaluation Research (iHope international), Japan.

## Results

A total of 736 patients participated in the study, of whom 687 (362 from clinics and 325 from hospitals) had complete data of the types of institutions where they received their prescription. Characteristics of study participants are shown in Table 1. The mean age of participants from clinics and hospitals was similar: 65.0 years. There was not a significant difference in the distribution of participants' educational attainment. A higher percentage of female patients was seen at clinics (53.3% vs. 43.3%, p = 0.01), and had fewer hypertensive complications than those seen at hospitals (p < 0.001). There was also a significant difference in the characteristics of patient-physician relationship (Table 2). The majority of the patients in both clinics and hospitals visited their physician once a month, but patients in clinics were seen more frequently than those in hospitals (p < 0.001). Patients seen at clinics tend to have a longer relationship with their personal physician (p = 0.001).

**Table 1:** Characteristics of study participants

	Type of institutions		
		
	Clinic (n = 362*)	Hospital (n = 325*)	
		
Characteristic	n (%)	n (%)	p value
Age			
Mean (SD)	65.0 (10.2)	65.0 (11.3)	0.945
Range	30-91	28-95	
Gender			
Male	164 (46.7)	178 (56.7)	0.010
Female	187 (53.3)	136 (43.3)	
Education			
<12 years	55 (15.9)	60 (19.4)	0.165
12 years	166 (48.1)	123 (39.4)	
>12 years	114 (33.0)	119 (38.4)	
Did not answer	10 (2.9)	8 (2.6)	
Complications of hypertension			
Retinal hemorrhage/detachment	20 (6.4)	33 (12.3)	0.014
Ischemic heart disease	32 (10.4)	58 (21.2)	<0.001
Stroke	16 (5.1)	29 (10.8)	0.011
Impaired renal function	23 (7.7)	46 (17.6)	<0.001
Any of the above	49 (17.1)	84 (34.7)	<0.001

* The numbers of each item do not sum up to the total because there were some missing or invalid data for each question.

**Table 2:** Characteristics of patient-physician relationship

	Type of institutions		
		
	Clinic (n = 362*)	Hospital (n = 325*)	
		
Characteristic	n (%)	n (%)	p value
Frequency of the visits			
> once a month	59 (17.3)	22 (7.1)	<0.001
once a month	215 (63.0)	160 (51.6)	
once in 2 months	27 (7.9)	89 (28.7)	
once in 3 months or more	40 (11.7)	39 (12.6)	
Length of the relationship			
less than a year	39 (11.3)	62 (19.7)	0.001
1-2 years	69 (19.9)	72 (22.9)	
3-5 years	99 (28.6)	96 (30.5)	
≥6 years	139 (40.2)	85 (27.0)	

* The numbers of each item do not sum up to the total because there were some missing or invalid data for each question.

Patients' perceptions of how well their physicians know their history and life circumstances are shown in Table 3. The six items were displayed in descending order of response as "knows very well or well." As for the patients' complete list of current medications, 79.3% of the patients said their physicians knew their list of current medications well, which was the highest of all the six aspects assessed. The lowest was the roles and responsibilities at work, home, or school: 28.3% answered "knows very well or well." When we compared the responses of those from clinics and hospitals, significant differences were observed in patients' worries about their own health (40.5% vs.29.3%, p = 0.004) and their roles and responsibilities at work, home, or school (32.2% vs. 24.0%, p = 0.028).

**Table 3:** Patients' perceptions of how well their physicians know about their history and life circumstances

	Type of institutions		
		
	Clinic (n = 362*)	Hospital (n = 325*)	
		
Physician's knowledge on patients'**:	n (%)	n (%)	p value
Complete list of current medication			
Knows very well, or well	248 (76.8)	243 (82.1)	0.103
Knows some, little, or not at all	75 (23.2)	53 (17.9)	
Entire past medical history			
Knows very well, or well	163 (48.4)	142 (47.5)	0.742
Knows some, little, or not at all	171 (51.2)	157 (52.5)	
History of allergy to drugs and food			
Knows very well, or well	136 (44.7)	136 (49.3)	0.274
Knows some, little, or not at all	168 (55.3)	140 (50.7)	
What worries patients most about their health			
Knows very well, or well	124 (40.5)	83 (29.3)	0.004
Knows some, little, or not at all	182 (59.5)	200 (70.7)	
Values and beliefs on health			
Knows very well, or well	102 (33.0)	89 (31.4)	0.685
Knows some, little, or not at all	207 (67.0)	194 (68.6)	
Roles and responsibilities at work, home, or school			
Knows very well, or well	98 (32.2)	67 (24.0)	0.028
Knows some, little, or not at all	206 (67.8)	212 (76.0)	

* The numbers of each item do not sum up to the total because there were some missing or invalid data for each question.

**The items were listed in the descending order of the percentage of the all patients answered "knows very well or well."

The results of the logistic regression analyses are shown in Table 4. In the multivariate model, the type of institutions (clinic/hospital) remained as a significant variable only for the patients' worries about their own health. The length of the patient-physician relationship significantly affected physicians' knowledge of past medical history, values and beliefs on health, and roles and responsibilities at work, home, or school. The factor that consistently affected all aspects except for the complete list of current medication was patients' age. Patients' gender did not affect any of the aspects (data not shown).

**Table 4:** Multivariate logistic regression analysis of the factors that influence patients' perception

Explanatory variables	Odds*	95% CI
	Complete list of current medication (Knows very well, or well)	
	
Clinic/Hospital	0.86	0.54-1.39
Frequency (<once/≥once in a month)	0.75	0.46-1.22
Length (≥3 years/<3 years)	0.94	0.58-1.51
Complications (one or more/none)	1.48	0.85-2.56
Patient's age (5 years)	1.03	0.93-1.14
	Entire past medical history (Knows very well, or well)	
	
Clinic/Hospital	0.97	0.65-1.44
Frequency (<once/≥once in a month)	0.86	0.57-1.31
Length (≥3 years/<3 years)	2.28	1.53-3.41
Complications (one or more/none)	1.69	1.09-2.60
Patient's age (5 years)	1.13	1.03-1.23
	History of allergy to drugs and food (Knows very well, or well)	
	
Clinic/Hospital	0.73	0.49-1.09
Frequency (<once/≥once in a month)	0.56	0.36-0.85
Length (≥3 years/<3 years)	1.43	0.96-2.13
Complications (one or more/none)	1.57	1.01-2.43
Patient's age (5 years)	1.11	1.02-1.22
	What worries patients most about their health (Knows very well, or well)	
	
Clinic/Hospital	1.54	1.02-2.35
Frequency (< once/≥once in a month)	0.69	0.44-1.09
Length (≥3 years/<3 years)	1.31	0.86-2.00
Complications (one or more/none)	1.13	0.71-1.78
Patient's age (5 years)	1.19	1.08-1.31
	Values and beliefs on health (Knows very well, or well)	
	
Clinic/Hospital	1.03	0.68-1.57
Frequency (<once/≥once in a month)	0.86	0.55-1.35
Length (≥3 years/<3 years)	1.61	1.04-2.48
Complications (one or more/none)	1.24	0.78-1.95
Patient's age (5 years)	1.12	1.01-1.23
	Roles and responsibilities at work, home, or school (Knows very well, or well)	
	
Clinic/Hospital	1.40	0.90-2.18
Frequency (<once/≥once in a month)	0.80	0.50-1.29
Length (≥3 years/<3 years)	1.86	1.17-2.94
Complications (one or more/none)	1.51	0.95-2.43
Patient's age (5 years)	1.13	1.02-1.25

*Odds were calculated as that of A/B for each of the first 4 variables

## Discussion

This study found that the difference of the type of medical institutions had little impact on the patients' perception of physicians' understandings of their background (Tables 3, 4). There was only one exception: whether patients were seen at clinics or hospitals significantly affected the knowledge of what patients worry about most regarding their health. Patients seen at clinics may consider that they can talk about their health concerns more easily with the physicians than those seen at hospitals. When interpreting this result, however, we should be aware that there may have been other confounders which were not adjusted for.

On the other hand, for aspects such as values and beliefs on health and roles and responsibilities at work, home, or school to be understood well, we found that patients might consider a longer relationship with physicians necessary. The length of the patient-physician relationship (i.e., continuity of care by the same personal physician) has been reported to be associated positively with the comprehensiveness of care [18] and patients' trust in their physicians [19]. Moreover, it is known to be related to patients' satisfaction with the care received [20-22], which is one component of outcomes of care [23]. It could be implied that, whether they see their patients at clinics or hospitals, a physician's whole-person knowledge of their patients, gained by maintaining the relationship would increase the patients' trust in their physician and their satisfaction with the care provided.

We also found that as patients aged, their assessment of physicians' whole-person knowledge became more favorable. The reason may be that older patients prefer visits with longer consultation time and in which they are asked to express their concerns [24]; thus, they might consider that they had provided personal information to their physicians more often than younger ones did. This finding is seen as consistent with the previous findings that older patients valued continuity of care by the same personal physician [25] and showed greater satisfaction with their physicians than did younger patients [26].

Our results showed a significant difference between the demographics of patients in clinics and hospitals; the percentage of female patients was higher and fewer patients had hypertensive complications. Patients at clinics saw their own physicians at shorter intervals and for more years. It could be inferred that clinics have a greater role in providing primary care in regard to care accessibility and availability for common and less severe conditions.

There are some limitations in this study. First, the generalizability of our results may be limited. As we do not know the refusal rate of participants of each group, our participants might not be fully representative of the patients with hypertension who are seen at clinics and hospitals in Japan. Besides, there are few pharmacies and their distribution is limited. Including patients with other chronic conditions such as diabetes or asthma would provide more general information about the quality of the primary care. Second, we only measured physicians' knowledge according to patients' perceptions. It was not a direct evaluation of physicians' knowledge, and results could have been influenced by patients' subjective evaluation of their own physicians. However, the measurement of patients' perception has been seen recently to be significant in assessing the quality of the primary care they receive [27]. Other indicators, such as patient-physician communication and provision of preventive counseling should be measured to arrive at a more thorough assessment of the quality of care. Third, when we approached patients in pharmacies, we were not able to identify the specialties of their prescribing physicians. Our results should not be interpreted as a comparison of the care provided by specialists and primary care physicians.

Although our study has several limitations described above, it still implied that Japanese physicians in hospitals and clinics have a common role in primary care for patients with hypertension. This might arise from the inadequacy of the primary care training for specialist physicians when they start working at clinics [7]. In 2006, the Japanese Academy of Family Medicine - now integrated to the Japan Primary Care Association - issued the first official requirements for residency programs to train family physicians [28]. Further discussion is needed on ways to retrain physicians in clinics in order to improve the quality of primary care in Japan.

## Conclusions

Hypertensive patients' perceptions of their physicians' knowledge about them did not differ significantly between clinics and hospitals in Japan, except for patients' worries about their health. Length of the relationship and patients' age had some impact on patients' perception of their physicians' knowledge. In order to differentiate the roles of physicians in hospitals and clinics more clearly and ensure the quality of primary care, the establishment of a standardized educational system to raise better trained primary care physicians is recommended.

## Competing interests

The authors declare that they have no competing interests.

## Authors' contributions

MI helped design the study, performed the statistical analysis, and drafted the manuscript. KI made substantial contributions to data interpretation and critically reviewed the manuscript. SM conceived and designed the study and made substantial contributions in its coordination. All authors read and approved the final manuscript.

## Pre-publication history

The pre-publication history for this paper can be accessed here:

http://www.biomedcentral.com/1471-2296/11/56/prepub

## Supplementary Material

Additional file 1 **Questionnaire**. The questionnaire translated into English. Click here for file
