# Supplementary material for: Hypertensive patients' perceptions of their physicians' knowledge about them: a cross-sectional study in Japan
Source: BMC Fam Pract. 2010 Aug 2;11:56. doi: 10.1186/1471-2296-11-56 (PMC2919452; doi:10.1186/1471-2296-11-56)
Supplement: Additional file 1 — Questionnaire. The questionnaire translated into English. [file 1471-2296-11-56-S1.PDF]

A survey on your prescribed drugs, your physician and pharmacist

Questionnaire

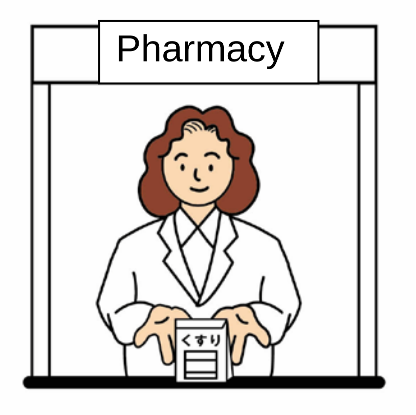

Ⅰ Please answer the following questions regarding your prescribed drugs.

Ⅰ－① How many kinds of prescribed drugs do you take regularly?

	kinds

Ⅰ－② How many physicians do you see regularly to be prescribed those drugs? Circle the number that corresponds to your response. (When you see more than two physicians in a hospital, count each one distinctively; ex. if you are seen and prescribed by an internist and an orthopedic surgeon, it will be two.)

**1**	One
**2**	Two
**3**	Three
**4**	Four or more

Ⅰ－③ How many kinds of drugs do you regularly take for **hypertension** (high blood pressure(BP)) ? Circle the number that corresponds to your response.

**1**	One
**2**	Two
**3**	Three or more

Ⅰ－④ Please write down the name(s) of your drug(s) for hypertension.

■ If you take more than three kinds of drugs, write about only the principal three of them.

■ How are you instructed to take the drug(s)? Circle the number that corresponds to the way you take each of them.

■ If you don't know the answer, leave it blank and proceed to the next question.

	Name of the drug	How you take them: Once a day	Twice a day	Three times a day	Only when the blood pressure is high
First	→	1	2	3	4
Second	→	1	2	3	4
Third	→	1	2	3	4

Ⅰ－⑤ Do you know what kind of side effects could occur by the drugs you take for hypertension? Circle the number that corresponds to your response.

1	I know all of them.
2	I know some of them.
3	I know little of them.
4	I don't know at all.

Ⅰ－⑥ Do you take your drugs for hypertension as instructed by your physician or pharmacist? Circle the number that corresponds to your response.

1	I always take drugs as instructed.
2	I usually take drugs as instructed.
3	It depends on the occasion.
4	I rarely take drugs as instructed.
5	I don't take drugs as instructed at all.

Ⅰ－⑦ Please read the following statements regarding hypertension (high BP). Circle the number that corresponds to your response to each statement.

	It is right	It is wrong	I don't know
■ It is not recommended to start taking a drug for hypertension when you had only one readings of high BP, as the readings of BP vary so often.	1	2	3
■ It is no use to treat senior patients with high BP because drug(s) don't extend their life.	1	2	3
■ Lowering the BP by taking drug(s) has a preventive effect of stroke (brain attack).	1	2	3
■ If you forget to take a dose of medicine, you can take it later together with another dose.	1	2	3
■ If the BP is not high, you can stop taking drug(s) by your own judgment.	1	2	3

Ⅱ Followings are the questions regarding your physician who prescribed drug(s) for hypertension today.

Ⅱ－① How long have you been seen by this physician?

1	Shorter than one month
2	One to less than six months
3	Six months to less than one year
4	One year to two years
5	Three to five years
6	Six to ten years
7	More than ten years

Ⅱ－② How often do you see this physician?

1	More than once in a week
2	Once in two weeks
3	Once in a month
4	Once in two months
5	Once in three months
6	Once in four months or longer

Ⅱ－③ What is this physician's specialty? Circle the number that corresponds to your response.

1	General internal medicine, Family medicine
2	Subspecialist in internal medicine; e.g. specialist of heart, digestive system, etc.
3	Specialist other than internal medicine; e.g. general surgeon, orthopedic surgeon etc.
4	I don't know

Ⅱ－④ What is your physician’s age approximately?

1	21－30 years old
2	31－40 years old
3	41－50 years old
4	51－60 years old
5	61－70 years old
6	71 years old or more
7	I don’t know

Ⅱ－⑤ How much do you think your physician knows about your history and life circumstances? Circle the number that corresponds to your response to each item.

	knows very well	knows well	knows to some extent	doesn’t know well	knows little	doesn’t know at all
■ Your past medical history and treatment	1	2	3	4	5	6
■ The entire list of your current medication	1	2	3	4	5	6
■ Your history of allergy to drugs and food	1	2	3	4	5	6
■ Your roles and responsibilities at work, home, or school	1	2	3	4	5	6
■ What worries you most about your health	1	2	3	4	5	6
■ Your values and beliefs on health	1	2	3	4	5	6

Ⅲ Followings are the questions regarding the pharmacy that filled your prescription today.

Ⅲ－① Is this the pharmacy do you usually get your prescription filled?

1	Yes
2	No

Ⅲ－② Have you ever received a written material about drug information at this pharmacy?

1	Yes
2	No
3	I don't remember

Ⅲ－③ What do you think of the pharmacist's explanation about your drug(s)?

1	Very good
2	Good
3	Fair
4	Not very good
5	Poor

Ⅲ－④ When you have any queries about your drug(s), who would you like to go? Circle the number that corresponds to your response to each of the items.

	Physician	Pharmacist	Both physician and pharmacist	Other than physician or pharmacist
■ Queries about the effect of the drug(s)	1	2	3	4

■	Queries about instructions and precautions for taking drugs	1	2	3	4
■	Other queries about drugs	1	2	3	4

Ⅳ Lastly, followings are the questions about yourself.

Ⅳ－① What is your age?

[ ] years old

Ⅳ－② What is your gender?

1	Male
2	Female

Ⅳ－③ What is the last school you finished?

1	Primary or secondary school
2	High school
3	Junior college or vocational school
4	College, university, or graduate school
5	I don't want to answer

Ⅳ－④ Have you ever had any of the following conditions? Circle the number that corresponds to your response to each of the items.

	Yes	No	I don't know
a) Retinal hemorrhage / detachments	1	2	3
b) Heart attack, angina	1	2	3
c) Stroke (brain attack)	1	2	3
d) Decline of kidney function	1	2	3

----- This is the end of the questionnaire. -----
